# Endo-Lysosomal Vesicles Positive for Rab7 and LAMP1 Are Terminal Vesicles for the Transport of Dextran

**DOI:** 10.1371/journal.pone.0026626

**Published:** 2011-10-24

**Authors:** William H. Humphries, Craig J. Szymanski, Christine K. Payne

**Affiliations:** School of Chemistry and Biochemistry and Petit Institute for Bioengineering and Bioscience, Georgia Institute of Technology, Atlanta, Georgia, United States of America; University of Nebraska Medical Center, United States of America

## Abstract

The endo-lysosomal pathway is essential for intracellular transport and the degradation of extracellular cargo. The relationship between three populations of endo-lysosomal vesicles—Rab7-positive, LAMP1-positive, and both Rab7- and LAMP1-postive—was probed with fluorescence microscopy and single particle tracking. Of specific interest was determining if these vesicles were intermediate or terminal vesicles in the transport of extracellular cargo. We find that the major organelle in the endo-lysosomal pathway, both in terms of population and cargo transport, is positive for Rab7 and LAMP1. Dextran, a fluid phase cargo, shifts from localization within all three populations of vesicles at 30 minutes and 1 hour to primarily LAMP1- and Rab7/LAMP1-vesicles at longer times. This demonstrates that LAMP1- and Rab7/LAMP1-vesicles are terminal vesicles in the endo-lysosomal pathway. We tested two possible mechanisms for this distribution of cargo, delivery to mannose 6-phosphate receptor (M6PR)-negative vesicles and the fusion dynamics of individual vesicles. We find no correlation with M6PR but do find that Rab7-vesicles undergo significantly fewer fusion events than LAMP1- or Rab7/LAMP1-vesicles suggesting that the distribution of fluid phase cargo is driven by vesicle dynamics.

## Introduction

The endo-lysosomal pathway is of fundamental importance in cell biology, responsible for the transport and degradation of extracellular cargo [1]–[4]. The conventional picture of the lysosomal degradation of extracellular cargo describes internalization of cargo from the plasma membrane, transport from early to late endosomes, and delivery of cargo to the lysosome, an acidic, enzyme-rich, membrane-bound organelle [5]–[7]. In recent years, a more complex picture of lysosomal degradation has emerged that demonstrates degradation can occur upstream of lysosomes and that key lysosomal proteins are not necessary for the degradation of extracellular cargo [8]–[13]. Reconciling these results with the conventional picture of the endo-lysosomal pathway has taken on increasing importance with the advent of gene delivery and nanobiotechnology, fields in which delivery of DNA or nanoparticles to enzyme-rich, degradative vesicles is either targeted for triggered release or avoided to prevent degradation [14], [15].

Understanding the endo-lysosomal pathway requires two steps. First, classifying endo-lysosomal vesicles based on their protein composition. Second, determining how extracellular cargo is transported by these vesicles. Recent results using two-color live cell imaging revealed three distinct populations of endo-lysosomal vesicles; Rab7-positive, lysosomal-associated membrane protein-1 (LAMP1)-positive, and vesicles positive for both Rab7 and LAMP1 [16]. We sought to determine the intertwined functions of these three populations of vesicles by examining the transport of extracellular cargo. As the transport of endocytic cargo is fundamentally dynamic, we have probed the endo-lysosomal pathway using multicolor single particle tracking fluorescence microscopy in addition to confocal microscopy. Of specific interest was determining the stage at which Rab7/LAMP1-vesicles enter the transport pathway. Are Rab7/LAMP1-vesicles intermediates between late endosomes and lysosomes or are they terminal vesicles in which cargo accumulates?

Dextran, a fluid phase marker [17]–[20], was fluorescently labeled and colocalization with each population of vesicle was measured. We find that at early times dextran is found in each type of vesicle, but ultimately accumulates in LAMP1- and Rab7/LAMP1-vesicles demonstrating that LAMP1- and Rab7/LAMP1-vesicles are terminal vesicles. We probed two possible mechanisms for the observed accumulation of dextran in LAMP1- and Rab7/LAMP1-vesicles. We classified the three populations of endo-lysosomal vesicles in terms of colocalization with the mannose 6-phosphate receptor (M6PR). Lysosomes, as compared to endosomes, are defined by the absence of M6PR [21]–[25]. As a second step, we probed the fusion dynamics of individual vesicles. We find no correlation with M6PR, but do observe fusion dynamics that support the observed partitioning of dextran.

## Results

We focused on two proteins, Rab7 and LAMP1, to classify the vesicles that compose the endo-lysosomal pathway. Rab7 is a GTPase most commonly associated with late endosomes [26]–[30]. While LAMP1 is typically considered lysosomal [31]–[34], it has also been reported to be late endosomal or pre-lysosomal [4], [22], [23], [35].

### Majority of endo-lysosomal vesicles are positive for Rab7 and LAMP1

**Figure 1:**
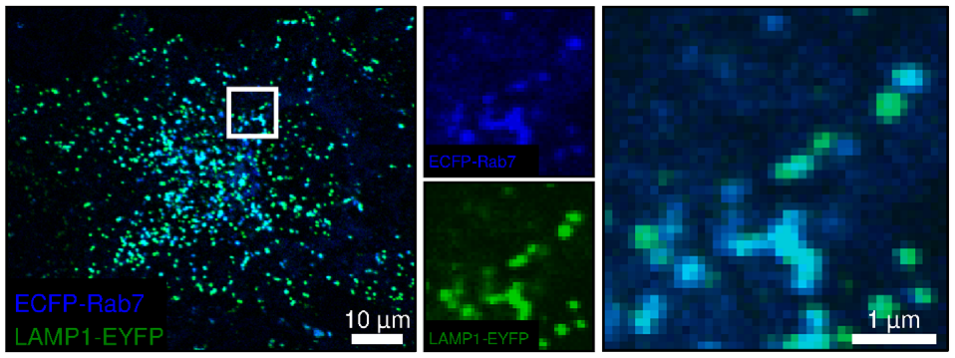
The majority of endo-lysosomal vesicles are positive for both Rab7 and LAMP1. A representative confocal microscopy image shows the overlaid ECFP-Rab7 (blue) and LAMP1-EYFP (green) images. The inset is split into its individual color components and enlarged. Similar levels of colocalization were observed for BS-C-1 cells stably expressing ECFP-Rab7, for endogenous Rab7 and LAMP1 in BS-C-1 cells, and for HeLa cells (Figure S1).

As previous work had suggested that the majority of endo-lysosomal vesicles are positive for both Rab7 and LAMP1 [16], we first determined what fraction of vesicles were positive for each protein combination. Confocal fluorescence microscopy was used to quantify the number of each type of vesicle in individual BS-C-1 monkey kidney epithelial cells (Figure 1). Rab7 and LAMP1 were labeled directly with the transient expression of spectrally-distinct fluorescent proteins; Rab7 with ECFP (blue) and LAMP1 with EYFP (green). The number of Rab7-, LAMP1- and Rab7/LAMP1-vesicles was determined manually by analyzing 10 random 100 µm^2^ regions of a cell. Values for the 10 regions were averaged and then scaled based on the total area of the cell. An analysis of 10 cells showed that the number of Rab7/LAMP1-vesicles ranged from 351 to 1288 depending on the individual cell. Rab7- and LAMP1-vesicles each represented ∼15% of the total endo-lysosomal vesicle population. Specifically, 12.7±3.5% of vesicles were positive for Rab7, but not LAMP1, and 14.4±5.1% of vesicles were positive for LAMP1, but not Rab7. The use of transient expression of ECFP-Rab7 and LAMP1-EYFP raises the possibility that the high percentage of vesicles positive for both Rab7 and LAMP1 is an artifact. To test this possibility, we measured the colocalization of Rab7 and LAMP1 in the absence of transient expression. Specifically, we used the same BS-C-1 cells as described above, but with stable, rather than transient, expression of ECFP-Rab7. Endogenous LAMP1 was detected with immunofluorescence. Similar levels of colocalization were observed with the majority of vesicles positive for both Rab7 and LAMP1 (Figure S1A). Additionally, the use of two-color immunofluorescence to detect Rab7 and LAMP1 revealed similarly high levels of colocalization in a completely endogenous system (Figure S1B). The high percentage of vesicles positive for both Rab7 and LAMP1 was not unique to BS-C-1 cells, but was also observed in HeLa cells (Figure S1C).

The relatively small number of Rab7- and LAMP1-vesicles relative to Rab7/LAMP1-vesicles raises two technical concerns. The first is that the single protein vesicles are only artifacts due to the specific labeling scheme used. To insure that these minority populations of Rab7- and LAMP1-vesicles were not artifacts, the same imaging methods were applied to cells with a different labeling scheme. Rab7 was fluorescently labeled with EYFP. The cells were then fixed and permeabilized. LAMP1-vesicles were labeled with a primary antibody against LAMP1 and a Cy5-labeled secondary antibody (Figure S2). Levels of colocalization similar to the Rab7-ECFP and LAMP1-EYFP labeling scheme were observed. The second concern is that the large number of Rab7/LAMP1-vesicles is due to cross-talk between emission channels; ECFP signal from Rab7 leaking through to the EYFP channel of LAMP1. For this reason, image analysis was carried out using carefully calibrated imaging parameters obtained from cells only expressing Rab7-ECFP or LAMP1-EYFP to prevent cross-talk between channels, as described in Materials and Methods. Additionally, the use of Rab7-EYFP and LAMP1 labeled with a Cy5 antibody (Figure S2), a fluorophore combination with less potential for cross-talk, resulted in similar levels of colocalization.

### Dextran accumulates in LAMP1- and Rab7/LAMP1-vesicles

Endo-lysosomal vesicles are defined not only by their protein composition, but also by their function, the transport of extracellular cargo. The lysosome has conventionally been defined as a terminal vesicle, the final stop on the endocytic pathway [7]. We sought to characterize Rab7-, LAMP1-, and Rab7/LAMP1-vesicles in terms of cargo transport; essentially using cargo to determine if Rab7/LAMP1-vesicles function as intermediates between late endosomes and lysosomes or as terminal vesicles, more similar to lysosomes.

**Figure 2:**
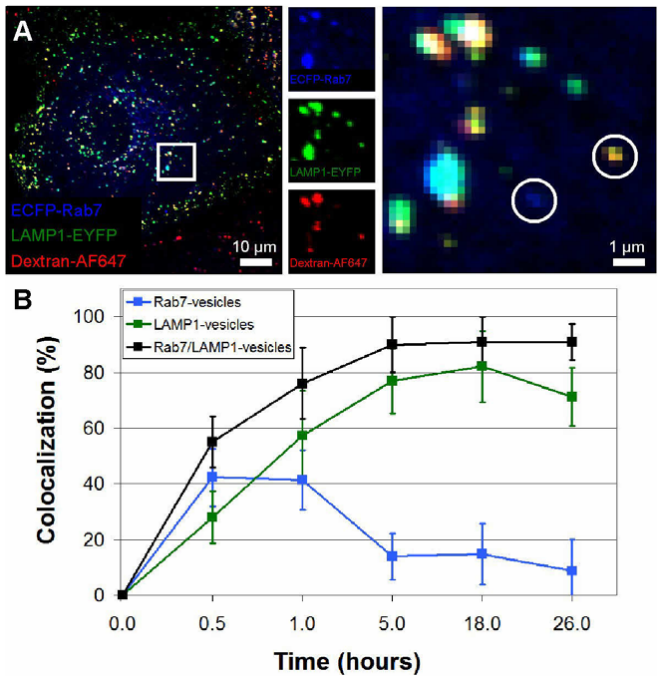
Dextran accumulates in LAMP1- and Rab7/LAMP1-vesicles. (*A*) Confocal microscopy image of ECFP-Rab7 (blue), EYFP-LAMP1 (green), and dextran-AF647 (red) after an 18 h incubation with dextran. The inset, split into its three color components and enlarged, shows a Rab7-vesicle in the absence of dextran and a LAMP1-vesicle containing dextran, both circled. (*B*) At early times dextran is present in Rab7- (blue), LAMP1- (green), and Rab7/LAMP1-vesicles (black). At longer times the percentage of Rab7-vesicles containing dextran decreases while the percentage of LAMP1- and Rab7/LAMP1-vesicles increase. The x-axis is plotted to highlight the early time data. Error bars represent standard deviation. At each time point, analysis was carried out for 10-15 vesicles per cell in 6–12 cells in 2–4 distinct experiments.

Dextran (10,000 MW) is a fluid-phase cargo, internalized by pinocytosis rather than receptor-mediated endocytosis [17]–[20]. Dextran was labeled with the red-emissive fluorophore AlexaFluor647 (AF647) and incubated with cells for either 30 min or 1 hr at a concentration of 0.25 mg/mL. For data recorded at 30 min or 1 hr, cells were fixed immediately following the incubation period. The data at 5 hr, 18 hr, and 26 hr were obtained from pulse-chase experiments. Cells were incubated with dextran for 1 hr, rinsed twice with complete cell culture media, and then incubated in dextran-free medium for 4 hr, 17 hr, and 25 hr. For image acquisition, cells were rinsed twice in PBS, fixed with 4% formaldehyde, and imaged with a confocal microscope (Figure 2). Ten to fifteen of each type of vesicle, in 6–12 cells, were randomly identified and manually scored for colocalization with dextran as described in Materials and Methods. After a 30 min incubation with dextran, 42±10% of Rab7-vesicles contained dextran. This level remained essentially the same (41±11%) after a 1 hr incubation and then decreased to 14±8% following a 4 hr chase. The decrease from 41% at 1 hr to 14% after a 4 hr chase is significant with a p-value less than 0.001. In comparison, 28±9% of LAMP1-vesicles contained dextran after a 30 min incubation. This value increased to 57±16% after a 1 hr incubation and remained high (>70%) at later times. After a 30 min incubation with dextran, 55±9% of Rab7/LAMP1-vesicles contained dextran. Like LAMP1-vesicles, this value increased after a 1 hr incubation (76±13%) and remained high (>90%) at all later time points.

### Colocalization of Rab7-, LAMP1-, and Rab7/LAMP1-vesicles with M6PR

**Figure 3:**
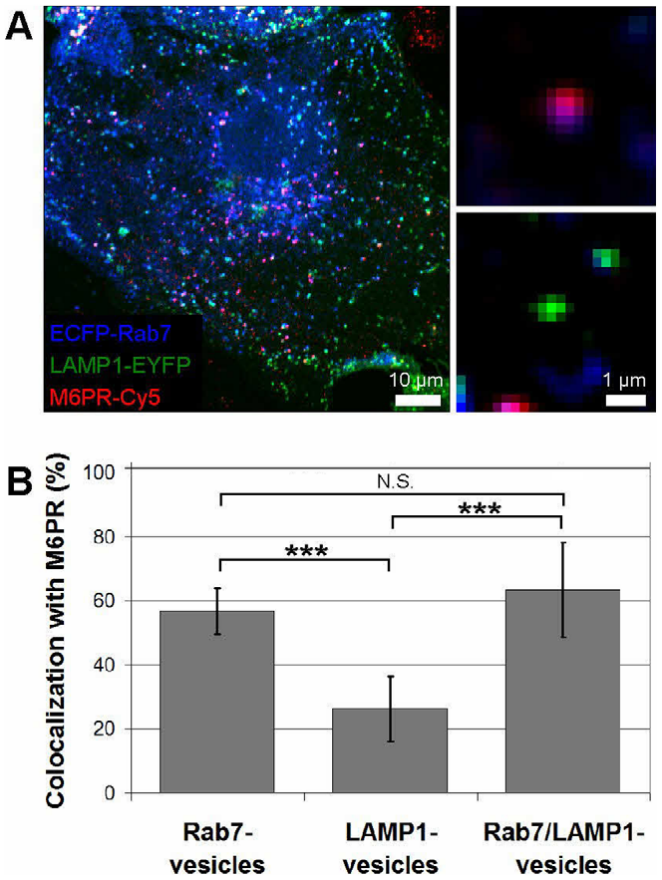
Colocalization of M6PR with Rab7-, LAMP1-, and Rab7/LAMP1-vesicles. (*A*) Confocal microscopy image of ECFP-Rab7 (blue), LAMP1-EYFP (green), and an antibody against M6PR labeled with a Cy5-labeled secondary antibody (red). Smaller images show a M6PR-positive (red) Rab7-vesicle (blue, top) and a M6PR-negative LAMP1-vesicle (green, bottom). (*B*) A large fraction of Rab7- and Rab7/LAMP1-vesicles are positive for M6PR; 57±7% and 63±15%, respectively. A smaller fraction of LAMP1-vesicles are positive for M6PR, 23±10%. Error bars show standard deviations. P-values<0.001 are indicated by ***. N.S. indicates a p-value >0.05. The graph shows the analysis of 10–20 of each type of vesicle per cell in 10 cells from 4 distinct experiments. Similar results were obtained for BS-C-1 cells stably expressing ECFP-Rab7 and for HeLa cells (Figure S4). Unmerged images are shown in Figure S5.

Confocal fluorescence microscopy was used to define Rab7-, LAMP1-, and Rab7/LAMP1-vesicles in terms of their colocalization with M6PR (Figure 3), the absence of which is often used to define lysosomes [21]–[25]. Rab7 and LAMP1 were labeled directly with ECFP (blue) and EYFP (green), respectively, as described above. M6PR was labeled with a primary antibody against M6PR and a Cy5-conjugated secondary antibody (red). Fixation, permeabilization, and imaging parameters are described in Materials and Methods. Colocalization was scored manually for 10 cells, examining 10–20 of each type of vesicle per cell. Fifty-seven percent of Rab7-vesicles were M6PR-positive as compared to 26% of LAMP1-vesicles. Sixty-three percent of Rab7/LAMP1-vesicles were positive for M6PR. Control experiments show that non-specific staining in the absence of the primary M6PR antibody is minimal (Figure S3) demonstrating that the observed colocalization is not an artifact. Additionally, the partial colocalization of M6PR with Rab7-, LAMP1-, and Rab7/LAMP1-vesicles was not unique to cells in which Rab7 was labeled through the transient expression of ECFP-Rab7. Similar levels of colocalization were observed for BS-C-1 cells in which Rab7 was labeled through the stable expression of ECFP-Rab7 (Figure S4A and B). HeLa human carcinoma cells also had levels of colocalization similar to BS-C-1 cells (Figure S4C and D).

### Fusion of Rab7-vesicles is rare

**Figure 4:**
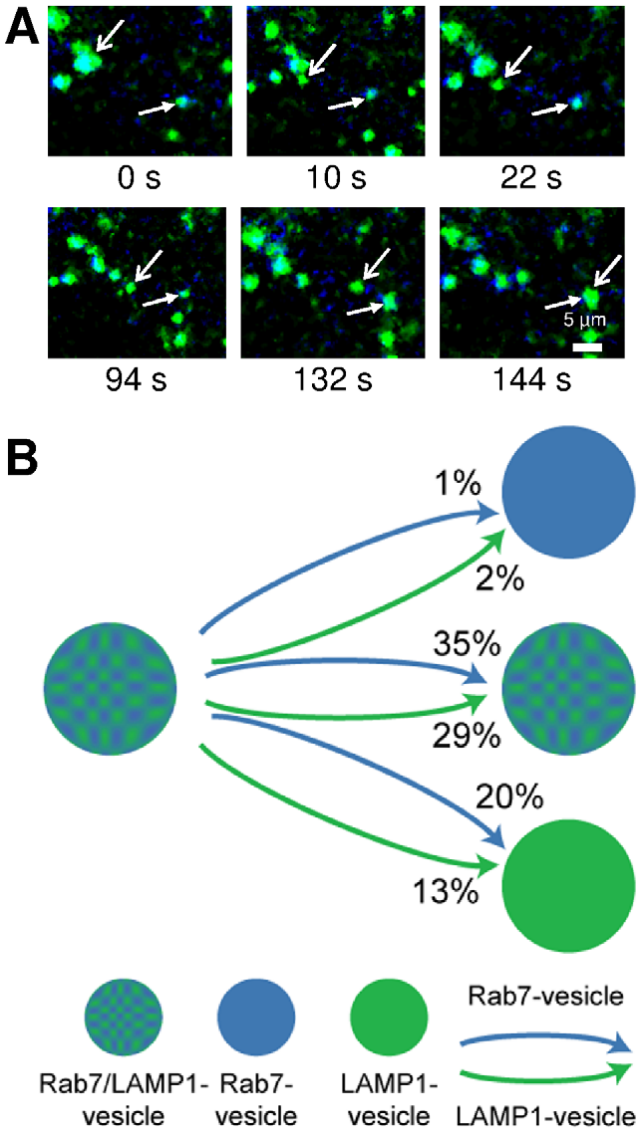
Single particle tracking fluorescence microscopy was used to follow the trajectories of Rab7- and LAMP1-vesicles. The trajectories of 50 Rab7-vesicles and 50 LAMP1-vesicles in 7 different cells were analyzed. (*A*) Snapshots of a trajectory obtained from two color live cell imaging experiments. These images show a LAMP1-vesicle (open arrow) split from a Rab7/LAMP1-vesicle, traffic through the cell, and then merge with a Rab7/LAMP1-vesicle (closed arrow). The corresponding movie (Movie S1) is included as supporting information. (*B*) The majority (89%) of trajectories began with a Rab7- (blue) or LAMP1-vesicle (green) splitting from a Rab7/LAMP1-vesicle (blue-green, patterned). The percentage of each type of event is shown. For example, 1% of Rab7-vesicles that split from a Rab7/LAMP1-vesicle then merged with another Rab7-vesicle.

To understand the partitioning of dextran, the interactions of the three vesicle populations were probed using two-color live cell imaging. Rab7 was labeled with ECFP (blue) and LAMP1 was labeled with EYFP (green). Cells were imaged using a multi-color microscope custom-built for live cell imaging and single particle tracking analysis. Images were recorded at a rate of 0.5 Hz for a period of 30 minutes with cells maintained at 37°C. Data, in the form of movies, were analyzed to detect Rab7- and LAMP1-vesicles and determine their interactions with other vesicles. Vesicles were considered Rab7/LAMP1-vesicles if the blue and green signals overlapped and moved together within the cell for a minimum of 10 s. An examination of 50 Rab7-vesicles and 50 LAMP1-vesicles in 7 cells showed that 100% of Rab7-vesicles and 78% of LAMP1-vesicles were initially part of a Rab7/LAMP1-vesicle. The Rab7- or LAMP1-vesicle appears to pinch off, traffic through the cell, and then fuse with another vesicle (Figure 4A). The majority of Rab7- and LAMP1-vesicles that were tracked from a Rab7/LAMP1-vesicle eventually merge with another Rab7/LAMP1-vesicle, 35% and 29%, respectively (Figure 4B). A smaller fraction of Rab7- and LAMP1-vesicles that split from a Rab7/LAMP1-vesicle merge with a LAMP1-vesicle, 20% and 13%, respectively. Fusion with a Rab7-vesicle was rarely observed.

## Discussion

Rab7 and LAMP1 are the two proteins most frequently used to define late endosomes and lysosomes, respectively [26]–[34], [36]–[38]. These definitions have been complicated by results that show Rab7-positive vesicles can carry out lysosomal functions including the degradation of extracellular cargo [10], [13]. Similarly, LAMP1 has been associated with late endosomes or pre-lysosomes [4], [22], [23], [35]. Lacking from previous work is an examination of Rab7 and LAMP1 simultaneously. Using confocal fluorescence microscopy to image Rab7 and LAMP1, we find that the majority (70%) of endo-lysosomal vesicles are positive for both Rab7 and LAMP1. This high percentage of Rab7/LAMP1-vesicles was not unique to the BS-C-1 cells (Figure 1), but was also observed in HeLa cells (91%, Figure S1C).

To probe the function of these three distinct populations of endo-lysosomal vesicles we measured the transport of dextran, a fluid-phase cargo, over relatively long time scales (30 min-26 hrs). Of specific interest was whether the Rab7/LAMP1-vesicles were intermediate, and do not retain cargo, or terminal, with the accumulation of cargo. After a 30 min incubation, dextran was found in all populations of vesicles with 55% of Rab7/LAMP1-vesicles containing dextran (Figure 2). At longer times, the percentage of LAMP1- and Rab7/LAMP1-vesicles containing dextran increases with >90% of Rab7/LAMP1-vesicles containing dextran after a 1 hr pulse and 4 hr chase. LAMP1-vesicles show a similar increase in the percentage containing dextran. In comparison, the percentage of Rab7-vesicles containing dextran decreases from 41% after a 1 hr incubation to ≤15% at longer times.

Terminal vesicles, in the absence of degradation, show an accumulation of cargo as a function of time as observed for LAMP1-vesicles. Interestingly, Rab7/LAMP1-vesicles behaved similarly to LAMP1-vesicles with an increase in dextran over time. In this sense, the Rab7/LAMP1-vesicle is best described as a terminal vesicle rather than an intermediate between late endosomes and lysosomes. The decrease of dextran in Rab7-vesicles as a function of time can be formally attributed degradation of dextran or the fluorophore, recycling to the plasma membrane, or transport to another endocytic vesicle [7]. The use of dextran as endocytic cargo limits these options as it is not degraded by the cell. As recycling is not expected to occur from the late endosome and the fluorophore is stable in the other vesicles, it is most likely that dextran is transported from Rab7-vesicles to LAMP1- and Rab7/LAMP1-vesicles although that is not measured directly in these experiments.

We focused on two possible mechanisms to understand the observed accumulation of dextran in LAMP1- and Rab7/LAMP1-vesicles. The first approach was to characterize each vesicle population in terms of the presence or absence of M6PR. M6PR, which is rapidly recycled from the lysosome [21]-[25], is commonly used to differentiate endosomes and lysosomes with lysosomes defined as M6PR-negative. We hypothesized that LAMP1- and Rab7/LAMP1-vesicles would show minimal colocalization with M6PR, as expected for lysosomes. Instead we found that the majority (63±15%) of Rab7/LAMP1-vesicles were positive for M6PR (Figure 3). A similar level of colocalization was observed in HeLa cells (Figure S4D). While the <100% colocalization of M6PR with Rab7-vesicles and the partial colocalization with LAMP1-vesicles has been described previously [22], [28], [37], [39], [40], the high colocalization of M6PR with Rab7/LAMP1-vesicles is a surprising result. Previous research using cryo-electron microscopy to follow the transport of cationized ferritin and horse radish peroxidase showed that LAMP (described as lgp120)-positive/M6PR-negative vesicles were terminal vesicles [22]. Instead, we find that Rab7/LAMP1-vesicles are terminal vesicles for the transport of dextran and are also M6PR-positive. The difference between our observation that M6PR-positive vesicles can serve as terminal vesicles and prior cryo-electron microscopy results is unclear, but may be due to differences in the specific cargo used as a marker for vesicle transport.

The second approach used to understand the partitioning of dextran was to examine the underlying dynamics and interactions of the three vesicle populations. The endo-lysosomal vesicles undergo repeated splitting and fusion events with all three types of vesicles splitting off and trafficking through the cell (Figure 4). We found that the majority of Rab7- and LAMP1-vesicles could be traced back to an origin at a Rab7/LAMP1-vesicle. The Rab7- and LAMP1-vesicles are highly mobile with transport speeds indicative of active transport carried out by motor proteins moving on the cytoskeleton [16]. We followed the trajectory of individual Rab7- and LAMP1-vesicles and noted their fusion partner. Interactions that led to the formation or maintenance of Rab7/LAMP1-vesicles represent the majority (86%) of interactions. The fusion of LAMP1-vesicles with other LAMP1-vesicles was also observed (13%), but the fusion of Rab7-vesicles with other Rab7-vesicles was rarely observed (1%).

The presence of Rab7 and LAMP1 on a single vesicle invites comparison to hybrid vesicles that have properties of both late endosomes and lysosomes. Hybrid vesicles have been observed directly using electron microscopy [22], [23], [41], [42], density gradient ultracentrifugation of cell-free endosomes and lysosomes [43], and functional assays probing the enzyme-mediated degradation of low-density lipoprotein (LDL) and ovalbumin within late endosomes [10], [13]. The Rab7/LAMP1-vesicles described in these experiments are hybrids in the sense that they are positive for both conventional late endosomal and lysosomal proteins. However they function as terminal vesicles rather than intermediates in the transport between late endosomes and lysosomes. This research may help to explain previous results, especially functional assays that have reported lysosomal activity associated with Rab7-positive vesicles, as the majority of these vesicles are likely Rab7/LAMP1-vesicles [10], [13].

In conclusion, we have found that the endo-lysosomal pathway is composed of at least three distinct populations of vesicles: Rab7-, LAMP1-, and Rab7/LAMP1-vesicles. The majority of endo-lysosomal vesicles are positive for both Rab7 and LAMP1. Measuring the location of dextran, a fluid phase cargo, as it moves through the endo-lysosomal pathway allows us to examine the role of Rab7/LAMP1-vesicles in the endo-lysosomal pathway. Dextran is present in Rab7-, LAMP1-, and Rab7/LAMP1-vesicles at early times, but shifts to primarily LAMP1- and Rab7/LAMP1-vesicles at later times demonstrating that LAMP1- and Rab7/LAMP1-vesicles are terminal vesicles in the endo-lysosomal pathway.

## Materials and Methods

### Cell culture

BS-C-1 and HeLa cells (both from ATCC, Manassas, VA) were maintained in a 37°C, 5% carbon dioxide environment in Minimum Essential Medium (MEM, Invitrogen, Carlsbad, CA) with 10% (v/v) fetal bovine serum (FBS, Invitrogen) and passaged every 3 days. For imaging, cells were cultured in 35 mm glass-bottom cell culture dishes (P35G-1.5-14-C, MatTek, Ashland, MA). For confocal imaging, cells were fixed with 4% formaldehyde for 30 minutes at room temperature then washed and imaged in PBS. For live cell imaging, cells were imaged in phenol red-free MEM (Invitrogen) buffered with 0.1 M HEPES to pH 8. The imaging medium was supplemented with 2% FBS, 1% glucose, and an oxygen scavenger (0.4 mg/mL glucose oxidase and 2 µL/mL catalase).

### Expression of fluorescently-labeled proteins

Endocytic vesicles were fluorescently labeled with the transient expression ECFP-Rab7 (a gift from S. Pfeffer) [26], EYFP-Rab7 (Addgene, 20164) [37], and LAMP1-EYFP (Addgene, 1816) [44]. Single transfections were carried out at a ratio of 4 µL FuGENE to 1–2 µg plasmid. Dual transfections were prepared by doubling the volume of FuGENE and using 1–2 µg of each plasmid. Experiments were carried out 24–48 h after transfection. The expression of these fluorescent proteins in BS-C-1 cells has been confirmed previously using colocalization with Rab9-EYFP and immunofluorescence for LAMP2 [10], [16], [37]. BS-C-1 cells stably expressing ECFP-Rab7 (Figure S6) were generated with the same ECFP-Rab7 plasmid and selection with G418 (345810, Calbiochem, Gibbstown, NJ).

### Internalization of dextran

Dextran-AF647 was purchased from Invitrogen (10,000 MW, fixable, D22914). Cells were incubated for 30 min or 1 h with 0.25 mg/mL dextran at 37°C in MEM supplemented with 10% FBS and then fixed at the specified time point.

### Immunofluorescence

Based on the method of J.X. Kang, et al. [45], cells for M6PR immunofluorescence were fixed with 4% formaldehyde for 30 min at room temperature and permeabilized (0.1% Triton-X 100 in PBS) for 5 min at room temperature. The primary antibody was added to cells at a 1:400 dilution in blocking buffer (10% FBS, 3% BSA in PBS) and incubated for 1 h at room temperature. The secondary antibody was added to cells at a 1:1000 dilution in blocking buffer and incubated for 30 min at room temperature. Cells were incubated in blocking buffer for 1 h prior to the addition of each antibody and washed in PBS three times between each step. The antibodies used in the course of these experiments were mouse cation-independent M6PR (MA1-066, Fisher Scientific) and Cy5 rabbit anti-mouse (AP160S, Chemicon, Temecula, CA). For LAMP1 immunofluorescence, cells were fixed with 2% formaldehyde for 40 min at room temperature and then permeabilized (3% BSA, 10% FBS, 0.5% Triton-X 100 in PBS) for 15 min at room temperature. Cells were incubated for 1 h in blocking buffer (10% FBS, 3% BSA in PBS) before the addition of antibody. The primary antibody, mouse LAMP1 (ab25630, Abcam, Cambridge, MA), was added to cells at a 2:1000 dilution in blocking buffer and incubated for 3–18 h at 4°C. The secondary antibody, Cy5 rabbit anti-mouse (AP160S, Chemicon), was added to cells at a 1:1000 dilution in blocking buffer and incubated for 30 min at room temperature. Cells were washed (0.3% BSA, 0.1% Triton-X 100 in PBS) three times between each step.

### Confocal microscopy

Confocal microscopy images were collected with a FluoView 1000 laser scanning confocal microscope (Olympus, Center Valley, PA) using a 1.42 N.A., 60x, oil immersion objective. ECFP was excited with a 405 nm diode laser, EYFP was excited with the 515 nm line of an argon ion laser and AF647 and Cy5 were excited by a 635 nm diode laser. For ECFP a 480–495 nm band pass filter was used to filter emission, for EYFP a 535–565 nm band pass filter, and for AF647 and Cy5 a 655–755 nm band pass filter was used. For all images, the pinhole was set to obtain a 1 µm thick optical slice.

### Single particle tracking fluorescence microscopy

For live cell imaging an inverted microscope (Olympus IX71) in an epi-fluorescent configuration with a 1.45 N.A., 60x, oil immersion objective (Olympus) was used to collect images. Excitation was supplied by two lasers: a tunable argon ion laser operating at 457 nm (35-LAP-431-208, Melles Griot, Carlsbad, CA) and a green diode (Green 532, Crystalaser, Reno, NV). Excitation beams were overlapped and focused on the back focal plane of the microscope objective. Cells were illuminated by both laser lines using a dichroic mirror (Z458/532/633RPC, Chroma). Excitation light was filtered from the emission by the appropriate filters: ECFP-Brightline 483/32 (Semrock, Rochester, NY) and EYFP-HQ580/50 (Chroma). Emission filters were mounted in a rotating filter wheel (FW103, Thorlabs, Newton, New Jersey). Images were recorded sequentially at a rate of 0.5 frames per second with a 200–300 ms exposure. The relatively fast switching time of the filter wheel (400 ms) in comparison to the motion of the vesicles (<1 µm/s) means that sequential imaging is equivalent to simultaneous imaging. Shutters (Uniblitz, Rochester, NY) limited exposure of the cells to the lasers. A full description can be found in Szymanski, et al [16]. Images were detected on an EMCCD camera (DU-888, Andor, South Windsor, CT). A heated stage plate and an objective heater were used to maintain the cells at 37°C during the course of experiments.

### Data analysis

ImageJ (http://rsb.info.nih.gov/ij/) was used for data analysis. Colocalization was scored manually using “Image5D” (http://rsb.info.nih.gov/ij/plugins/image5d.html). Vesicles for analysis were chosen randomly by overlaying each image with a 10 µm×10 µm grid. The vesicle closest to the center of the grid was used for analysis. Tracking was performed using the ImageJ measurement tool in combination with a frame-forwarding macro. Intensity levels for image analysis were determined by imaging cells labeled with single fluorophores and measuring the cross-talk between channels. Images for publication were background subtracted and intensities were adjusted equally within each data set. Significance testing was performed using a two-tailed Welch's t-test to obtain a p-value.

## Supporting Information

Figure S1 **Rab7 and LAMP1 are highly colocalized in BS-C-1 cells and in HeLa cells.** (A) A representative confocal microscopy image shows the overlay of ECFP-Rab7 (blue) from BS-C-1 cells stably expressing ECFP-Rab7 and LAMP1 (red), labeled with a primary antibody against LAMP1 (1:100, ab25630, Abcam, Cambridge, MA) and a Cy5-labeled secondary antibody (1:500, AP160S, Chemicon, Temecula, CA). Smaller images show the individual color components. We measured 85±7% colocalization of Rab7-vesicles with LAMP1-vesicles and 90±6% colocalization of LAMP1-vesicles with Rab7-vesicles. Colocalization values were calculated for 9–2 vesicles per cell for 10 cells in 2 distinct experiments. (B) A representative confocal microscopy image shows the overlay of endogenous Rab7 (red) and LAMP1 (blue). Smaller images show the individual color components. Rab7 was labeled with a primary antibody against Rab7 (1:100, 9367, Cell Signaling, Danvers, MA) and a Cy5-labeled secondary antibody (1:500, ab97077, Abcam). LAMP1 was labeled with the same primary antibody as described above and a FITC-labeled secondary antibody (1:500, ab7064, Abcam). We measured 85±8% colocalization of Rab7-vesicles with LAMP1-vesicles and 82±11% colocalization of LAMP1-vesicles with Rab7-vesicles. Colocalization values were calculated for 10–15 vesicles per cell for 10 cells in 2 distinct experiments. (C) A representative confocal microscopy image shows overlaid ECFP-Rab7 (blue) and LAMP1-EYFP (green) images resulting from transient expression in HeLa cells. Smaller images show the individual color components. We measured 91±6% colocalization of Rab7-vesicles with LAMP1-vesicles and 91±4% colocalization of LAMP1-vesicles with Rab7-vesicles. Colocalization values were calculated for 12–14 vesicles per cell for 5 cells in 2 distinct experiments.
(TIF)

Figure S2 **Similar levels of Rab7- and LAMP1-vesicle colocalization were observed with an alternate labeling scheme.** A confocal microscopy image of BS-C-1 cells in which Rab7 is labeled with EYFP and LAMP1 is labeled with a primary antibody against LAMP1 (ab25630, Abcam) and a Cy5-labeled secondary antibody (AP160S, Chemicon). The inset, split into its individual color components and enlarged, shows a Rab7-vesicle (green, circled). The colocalization of Rab7-vesicles with LAMP1-vesicles (89±4%) and the reverse (89±6%) were similar to those obtained with the ECFP-Rab7/LAMP1-EYFP labeling scheme. Colocalization values were calculated for 25 vesicles per cell for 9 cells in 3 distinct experiments.
(TIF)

Figure S3 **The colocalization of LAMP1- and Rab7/LAMP1-vesicles with M6PR is not due to non-specific binding.** (A) Confocal microscopy image showing the Cy5 emission from a BS-C-1 cell labeled with a primary antibody for M6PR and a Cy5-labeled secondary antibody. The corresponding three color image is shown in Figure 3A. (B) The Cy5 emission of a BS-C-1 cell with the same fixation, permeabilization, blocking, and imaging conditions in the absence of the primary M6PR antibody.
(TIF)

Figure S4 **Colocalization of M6PR with Rab7-, LAMP1-, and Rab7/LAMP1-vesicles in BS-C-1 cells stably expressing ECFP-Rab7 and in HeLa cells.** (A) Confocal microscopy image of ECFP-Rab7 (blue) from a BS-C-1 cell line stably expressing ECFP-Rab7, the transient expression of LAMP1-EYFP (green), and an antibody against M6PR (MA1-066, Fisher Scientific) labeled with a Cy5 secondary antibody (red, AP160S, Chemicon). (B) A significant fraction of Rab7-, LAMP1-, and Rab7/LAMP1-vesicles are positive for M6PR; 48±4%, 27±7%, and 52±12%, respectively. Error bars show standard deviations. The graph shows the analysis of 10 of each type of vesicle per cell in 9 cells. Similar results were obtained for BS-C-1 cells transiently expressing ECFP-Rab7, Figure 3. (C) Confocal microscopy image of ECFP-Rab7 (blue), LAMP1-EYFP (green), and an antibody against M6PR labeled with a Cy5 secondary antibody (red) in HeLa cells. (D) As with the BS-C-1 cells, a significant fraction of Rab7-, LAMP1-, and Rab7/LAMP1-vesicles are positive for M6PR; 39±13%, 33±4%, and 64±6%, respectively. Error bars show standard deviations. The graph shows the analysis of 10–15 of each type of vesicle per cell in 5 cells. P-values<0.001 are indicated by ***,<0.01 by **. N.S. indicates p-values >0.05.
(TIF)

Figure S5 **Single color confocal microscopy images from** **Figure 3** **.** (A) ECFP-Rab7 (blue). (B) LAMP1-EYFP (green). (C) Antibody against M6PR labeled with a Cy5 secondary antibody (red).
(TIF)

Figure S6 **Western blot of BS-C-1 cells stably expressing ECFP-Rab7**. BS-C-1 cells and BS-C-1 cells stably expressing ECFP-Rab7 were lysed in a 1% Triton X-100 lysis buffer containing a protease inhibitor (Halt, 78441, Pierce, Rockford, IL) for 30 min at 4°C followed by centrifugation at 14,000 rcf for 20 min at 4°C. BCA analysis was used to determine protein concentration. Lysate was diluted in a Laemmli loading buffer (BP-110R), run on a Tris-glycine SDS gel (456–1094, Bio-Rad, Hercules, CA), and transferred to a PVDF membrane. The membrane was blocked (Near IR Blocking Buffer, MB-070, Rockland Immunochemicals, Gilbertsville, PA) for 1 hr at room temperature. Primary antibodies were incubated overnight at 4°C in blocking buffer and the membrane was washed with TBS-Tween. Secondary antibodies were incubated for 2 hrs at room temperature in blocking buffer. Rab7 (1:1000, 9367, Cell Signaling) was detected with a secondary antibody labeled for emission at 700 nm (red, 1:10,000, 926–68021, LI-COR, Lincoln, NE). GAPDH (1:1000, ab9484, Abcam), detected with a secondary labeled for emission at 800 nm (green, 1:5000, 926–32212, LI-COR), was used as a loading control. The membrane was imaged with an Odyssey Imager (LI-COR). Rab7 (23 kDa), ECFP-Rab7 (50 kDa), and GAPDH (37 kDa) were present at their expected molecular weights.
(TIF)

Movie S1 **Movie corresponding to snapshots in** **Figure 4A** **.** The playback speed is 7 frames/second.
(AVI)
