# Supplementary figures and images for: Endo-Lysosomal Vesicles Positive for Rab7 and LAMP1 Are Terminal Vesicles for the Transport of Dextran
Source: PLoS One. 2011 Oct 24;6(10):e26626. doi: 10.1371/journal.pone.0026626 (PMC3200357; doi:10.1371/journal.pone.0026626)

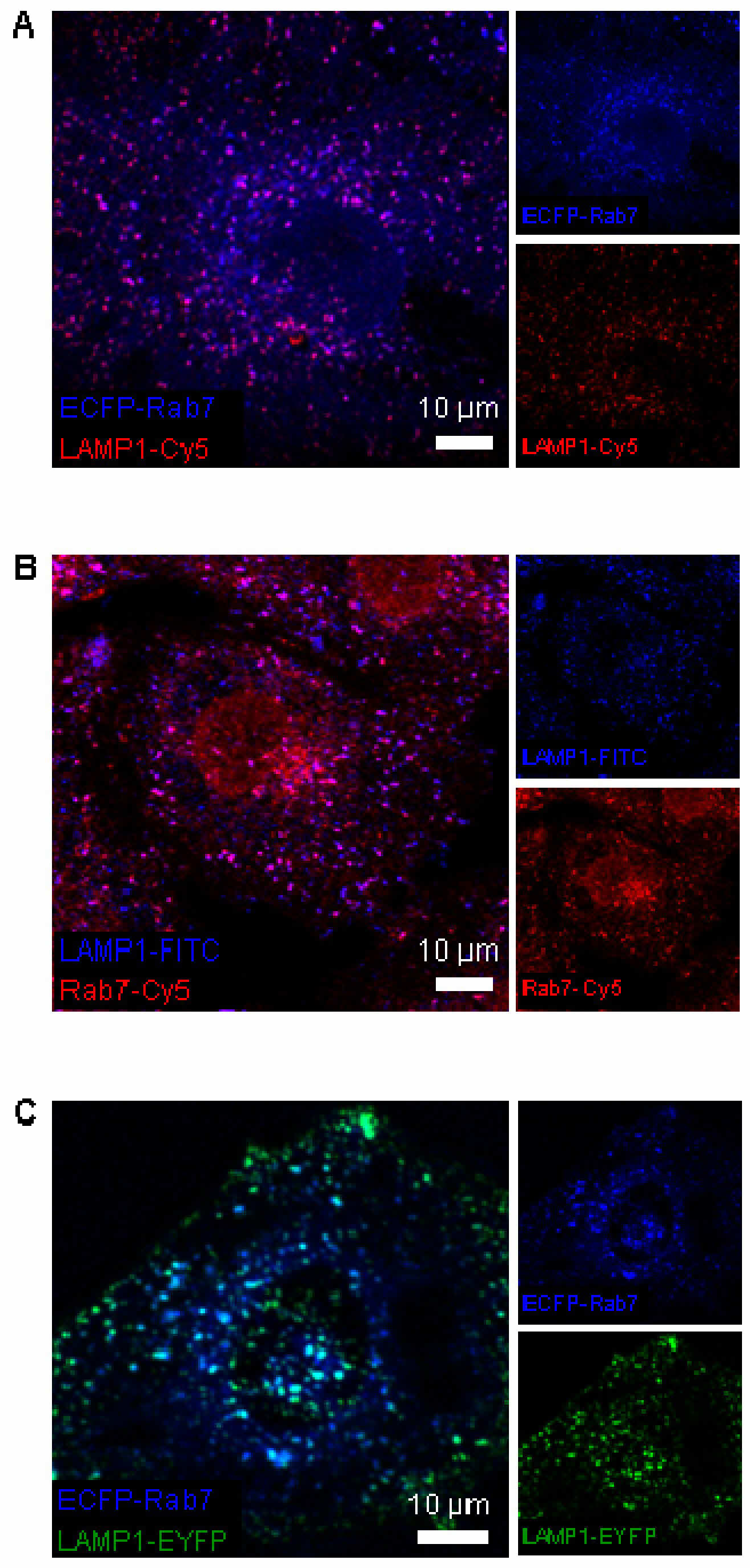

Supplement: Figure S1 — Rab7 and LAMP1 are highly colocalized in BS-C-1 cells and in HeLa cells. (A) A representative confocal microscopy image shows the overlay of ECFP-Rab7 (blue) from BS-C-1 cells stably expressing ECFP-Rab7 and LAMP1 (red), labeled with a primary antibody against LAMP1 (1:100, ab25630, Abcam, Cambridge, MA) and a Cy5-labeled secondary antibody (1:500, AP160S, Chemicon, Temecula, CA). Smaller images show the individual color components. We measured 85±7% colocalization of Rab7-vesicles with LAMP1-vesicles and 90±6% colocalization of LAMP1-vesicles with Rab7-vesicles. Colocalization values were calculated for 9–2 vesicles per cell for 10 cells in 2 distinct experiments. (B) A representative confocal microscopy image shows the overlay of endogenous Rab7 (red) and LAMP1 (blue). Smaller images show the individual color components. Rab7 was labeled with a primary antibody against Rab7 (1:100, 9367, Cell Signaling, Danvers, MA) and a Cy5-labeled secondary antibody (1:500, ab97077, Abcam). LAMP1 was labeled with the same primary antibody as described above and a FITC-labeled secondary antibody (1:500, ab7064, Abcam). We measured 85±8% colocalization of Rab7-vesicles with LAMP1-vesicles and 82±11% colocalization of LAMP1-vesicles with Rab7-vesicles. Colocalization values were calculated for 10–15 vesicles per cell for 10 cells in 2 distinct experiments. (C) A representative confocal microscopy image shows overlaid ECFP-Rab7 (blue) and LAMP1-EYFP (green) images resulting from transient expression in HeLa cells. Smaller images show the individual color components. We measured 91±6% colocalization of Rab7-vesicles with LAMP1-vesicles and 91±4% colocalization of LAMP1-vesicles with Rab7-vesicles. Colocalization values were calculated for 12–14 vesicles per cell for 5 cells in 2 distinct experiments. (TIF) [file pone.0026626.s001.tif]

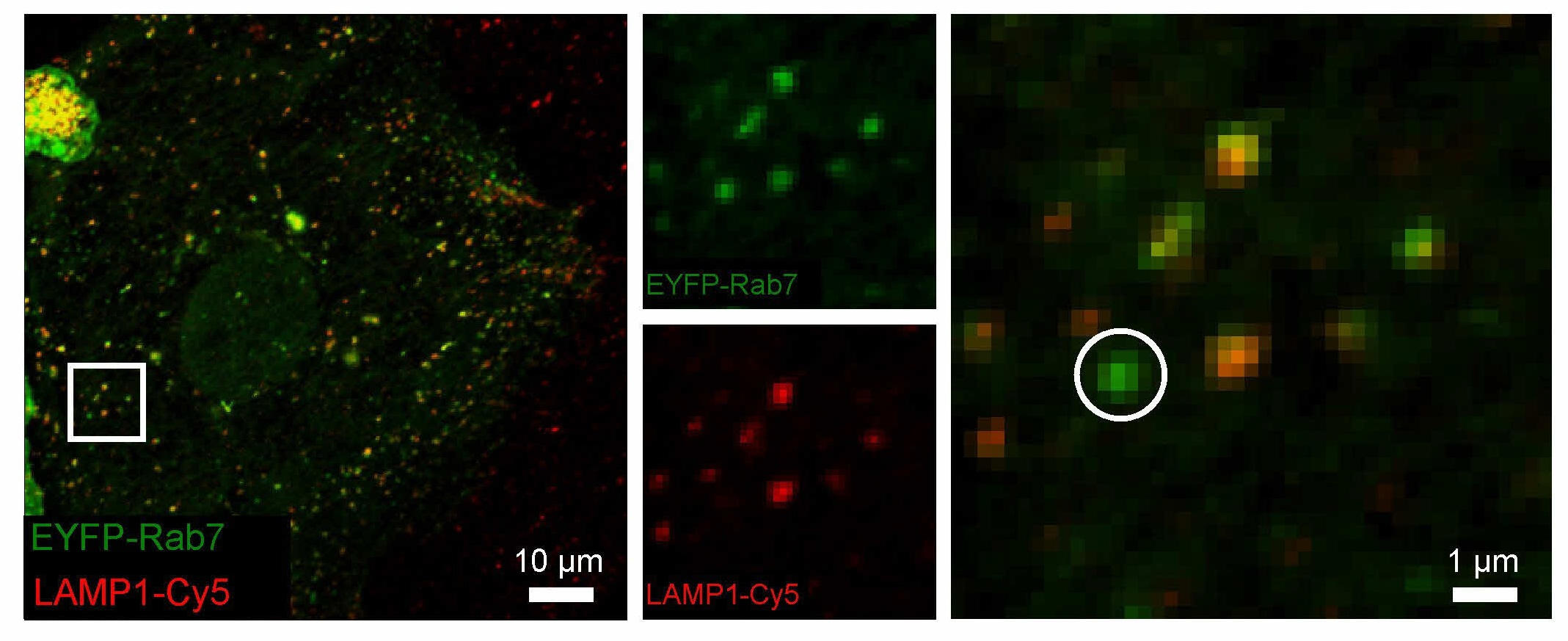

Supplement: Figure S2 — Similar levels of Rab7- and LAMP1-vesicle colocalization were observed with an alternate labeling scheme. A confocal microscopy image of BS-C-1 cells in which Rab7 is labeled with EYFP and LAMP1 is labeled with a primary antibody against LAMP1 (ab25630, Abcam) and a Cy5-labeled secondary antibody (AP160S, Chemicon). The inset, split into its individual color components and enlarged, shows a Rab7-vesicle (green, circled). The colocalization of Rab7-vesicles with LAMP1-vesicles (89±4%) and the reverse (89±6%) were similar to those obtained with the ECFP-Rab7/LAMP1-EYFP labeling scheme. Colocalization values were calculated for 25 vesicles per cell for 9 cells in 3 distinct experiments. (TIF) [file pone.0026626.s002.tif]

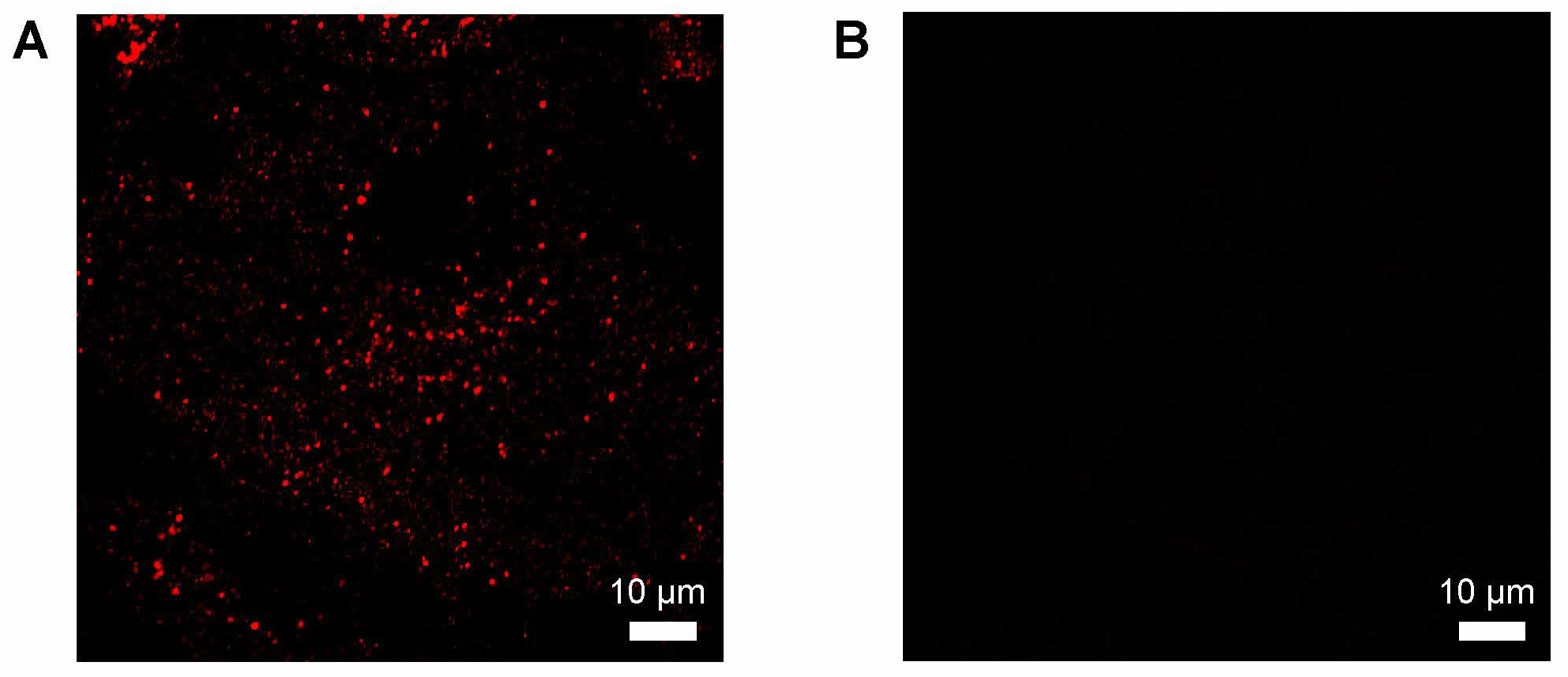

Supplement: Figure S3 — The colocalization of LAMP1- and Rab7/LAMP1-vesicles with M6PR is not due to non-specific binding. (A) Confocal microscopy image showing the Cy5 emission from a BS-C-1 cell labeled with a primary antibody for M6PR and a Cy5-labeled secondary antibody. The corresponding three color image is shown in Figure 3A. (B) The Cy5 emission of a BS-C-1 cell with the same fixation, permeabilization, blocking, and imaging conditions in the absence of the primary M6PR antibody. (TIF) [file pone.0026626.s003.tif]

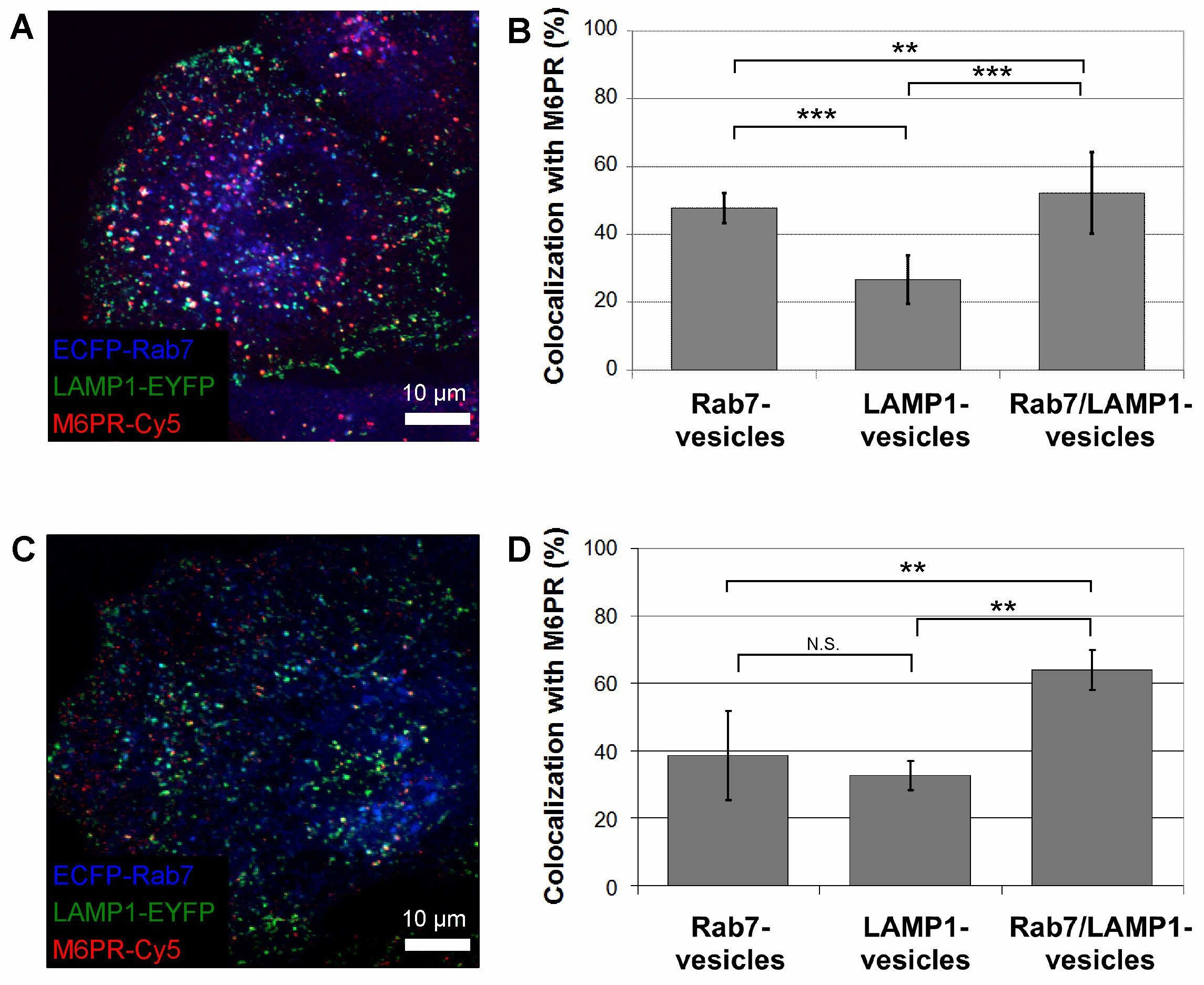

Supplement: Figure S4 — Colocalization of M6PR with Rab7-, LAMP1-, and Rab7/LAMP1-vesicles in BS-C-1 cells stably expressing ECFP-Rab7 and in HeLa cells. (A) Confocal microscopy image of ECFP-Rab7 (blue) from a BS-C-1 cell line stably expressing ECFP-Rab7, the transient expression of LAMP1-EYFP (green), and an antibody against M6PR (MA1-066, Fisher Scientific) labeled with a Cy5 secondary antibody (red, AP160S, Chemicon). (B) A significant fraction of Rab7-, LAMP1-, and Rab7/LAMP1-vesicles are positive for M6PR; 48±4%, 27±7%, and 52±12%, respectively. Error bars show standard deviations. The graph shows the analysis of 10 of each type of vesicle per cell in 9 cells. Similar results were obtained for BS-C-1 cells transiently expressing ECFP-Rab7, Figure 3. (C) Confocal microscopy image of ECFP-Rab7 (blue), LAMP1-EYFP (green), and an antibody against M6PR labeled with a Cy5 secondary antibody (red) in HeLa cells. (D) As with the BS-C-1 cells, a significant fraction of Rab7-, LAMP1-, and Rab7/LAMP1-vesicles are positive for M6PR; 39±13%, 33±4%, and 64±6%, respectively. Error bars show standard deviations. The graph shows the analysis of 10–15 of each type of vesicle per cell in 5 cells. P-values<0.001 are indicated by ***,<0.01 by **. N.S. indicates p-values >0.05. (TIF) [file pone.0026626.s004.tif]

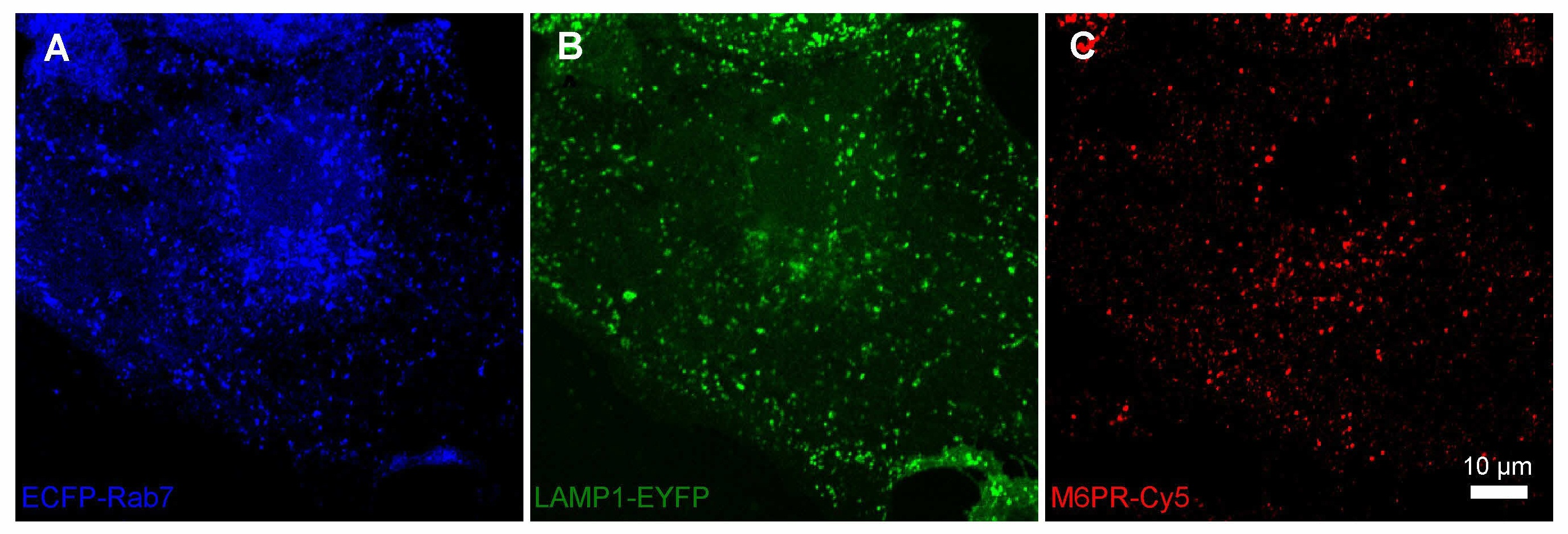

Supplement: Figure S5 — Single color confocal microscopy images from Figure 3 . (A) ECFP-Rab7 (blue). (B) LAMP1-EYFP (green). (C) Antibody against M6PR labeled with a Cy5 secondary antibody (red). (TIF) [file pone.0026626.s005.tif]

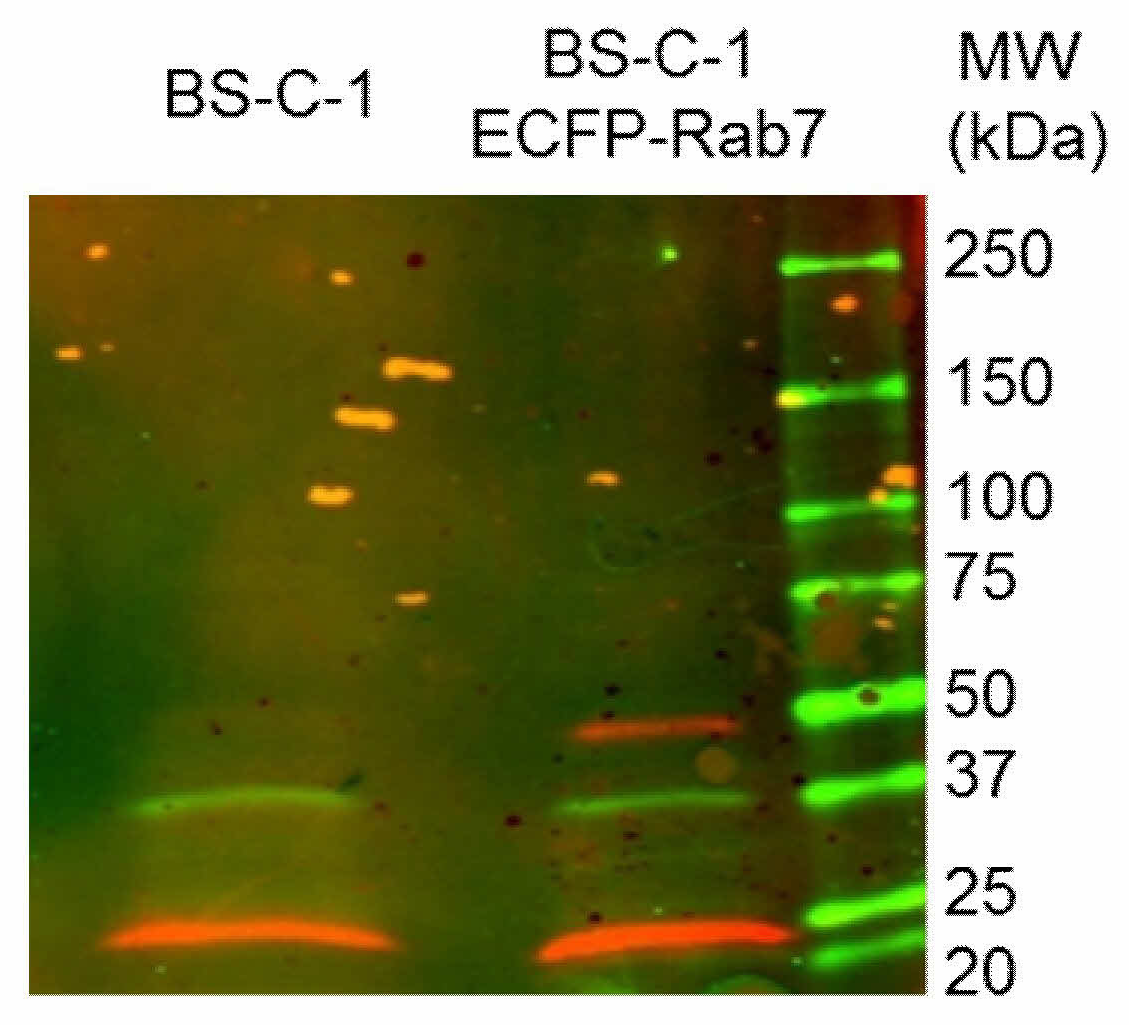

Supplement: Figure S6 — Western blot of BS-C-1 cells stably expressing ECFP-Rab7. BS-C-1 cells and BS-C-1 cells stably expressing ECFP-Rab7 were lysed in a 1% Triton X-100 lysis buffer containing a protease inhibitor (Halt, 78441, Pierce, Rockford, IL) for 30 min at 4°C followed by centrifugation at 14,000 rcf for 20 min at 4°C. BCA analysis was used to determine protein concentration. Lysate was diluted in a Laemmli loading buffer (BP-110R), run on a Tris-glycine SDS gel (456–1094, Bio-Rad, Hercules, CA), and transferred to a PVDF membrane. The membrane was blocked (Near IR Blocking Buffer, MB-070, Rockland Immunochemicals, Gilbertsville, PA) for 1 hr at room temperature. Primary antibodies were incubated overnight at 4°C in blocking buffer and the membrane was washed with TBS-Tween. Secondary antibodies were incubated for 2 hrs at room temperature in blocking buffer. Rab7 (1:1000, 9367, Cell Signaling) was detected with a secondary antibody labeled for emission at 700 nm (red, 1:10,000, 926–68021, LI-COR, Lincoln, NE). GAPDH (1:1000, ab9484, Abcam), detected with a secondary labeled for emission at 800 nm (green, 1:5000, 926–32212, LI-COR), was used as a loading control. The membrane was imaged with an Odyssey Imager (LI-COR). Rab7 (23 kDa), ECFP-Rab7 (50 kDa), and GAPDH (37 kDa) were present at their expected molecular weights. (TIF) [file pone.0026626.s006.tif]
